# Please do not feel bad, identifying the precise study design used in clinical research may be a challenge

**DOI:** 10.1002/cre2.211

**Published:** 2019-06-20

**Authors:** Asbjørn Jokstad

**Affiliations:** ^1^ Faculty of Health Sciences, UiT The Arctic University of Norway Tromsø Norway

Countless textbooks in epidemiology and medical statistics describe characteristics of descriptive and experimental clinical studies. Early postwar textbooks tended to focus on core elements and principles on how to plan and execute clinical research, and provided examples of how data from weak study methodologies have the potential to cause a range of fallacies and harm (Bradford Hill, 1971). The naming of the different strategies adopted to assemble study participants and accumulate data combined with the approaches to draw statistical inferences has come later. Unfortunately, varieties of different terms used to describe clinical research have emerged, which creates confusing terminology recognized two decades ago (Gordis, 1996) that is persistent even today (Celentano & Szklo, 2018). Moreover, multiple examples exist where the text does not reflect the alleged study design implemented in the title in the materials and methods section of the paper.

One major impetus for the need to characterize different clinical study designs was in the 60s to facilitate searching in emerging digital bibliographic databases, of which the predecessor of MEDLINE and PubMed, ie, MEDLARS, was the most important. Librarians could efficiently retrieve titles by combining so‐called Medical Subject Headings (MeSH) in Boolean expressions. Many authors today include in the title of their papers the MESH terms introduced in 1965 to describe persons, and not necessarily patients, to report outcomes or characteristics of patients that have received therapy. It is difficult and time‐consuming to assess the proportion of titles that reflects a study population consisting of patients that have received therapy amongst the papers where the title contains the term “prospective study” (*n* = 32,499 papers in PubMed), “retrospective study” (*n* = 22,249), and “follow‐up study”(*n* = 17,953). These terms became subheadings of “cohort studies” when this term was introduced as a MESH‐term in 1989. The term “case reports” (*n* = 15,778) was introduced as a type of publication in 1966 and does not require any description of outcomes of therapy but is only described as: “clinical presentations that may be followed by evaluative studies that eventually lead to a diagnosis.” (URL: https://www.ncbi.nlm.nih.gov/mesh/68002363).

“Case series” (*n* = 16,423) is an intriguing term for several reasons, although it has never been a MEDLINE MESH‐term or publication type. Many share today the understanding promoted by EBM protagonists (Guyatt, Sackett, & Haynes, 2006) and the National Cancer Institute that a case series denotes: “A group or series of case reports involving patients who were given similar treatment. Reports of case series usually contain detailed information about the individual patients. This includes demographic information (for example, age, gender, ethnic origin) and information on diagnosis, treatment, response to treatment, and follow‐up after treatment” (NCI, 2019). However, this term has had another interpretation that precedes the NCI explanation, which is to denote a descriptive study of individuals exposed to a known exposure, alternatively patients with disease followed in time. Fletcher and colleagues have authored an excellent textbook on clinical epidemiology and it is interesting to read the transition in their descriptions of how to interpret data observed in such “false cohorts” in, eg, Edition 3, p. 211; Edition 4, p. 114; and Edition 5, p. 101 (Fletcher & Fletcher, 2013). It is therefore understandable why attempts to clarify differences between descriptive and exploratory clinical studies appear regularly and authored by, for example, epidemiologists (Dekkers, Egger, Altman, & Vandenbroucke, 2012), neurologists (Esene et al., 2014), or veterinarians (Sargeant, O'Connor, Cullen, Makielski, & Jones‐Bitton, 2017). Further confounding is introduced by suggestions for the use of alternative terms to “case series.” Examples are “uncontrolled study” (*n* = 60 titles in PubMed; Altman, 1991), “uncontrolled trial” (*n* = 56; Groten, Janda, & Latta, 2004; Pocock, 1983), “single cohort study” (*n* = 30; Hulley & Cummings, 1988), or “case study” (Brunette, 1996), which otherwise is a research method common in social and life sciences. As we are fortunate not to live in an Orwellian society, we have access to old texts and it will remain a challenge to create consensus about appropriate terminology on study designs and compliance to their correct usage in titles and text.

The term cross‐sectional study has been adopted for studies in which the presence or absence of disease or other health‐related variables are determined in each member of the study population or a representative sample at one particular time. It is therefore intriguing to discover titles containing the descriptors “retrospective cross‐sectional” and “prospective cross‐sectional” studies. A rapid search that combines (“cross‐sectional”[ti] AND (“prospective”[ti] OR “retrospective”[ti])”) yields *n* = 1,166 papers, of which 32 can be found in dental journals by filtering for dental journals. It is rapidly obvious that different authors over time and place have had their different ideas for choosing to include the terms in their titles, and it takes some time to discriminate amongst the study objectives and methodologies.

One regular use is to describe meta‐analyses of cross‐sectional data over particular periods to appraise trends in the prevalence of diseases or health conditions such as allergy or preterm birth in subpopulations. It makes sense to describe these studies as, for example, repeated or serial cross‐sectional studies or retrospective cross‐sectional studies. Another use is to analyze trends in claims databases and health registries, albeit a host of biases may be identified. An alternative approach, which is perhaps more controversial, is to reflect how the recruitment of study participants to the cross‐sectional study has happened (Bacchieri & Della Cioppa, 2007). One has to keep in mind that in many situations, there is no access to patient records that may enable a methodologically better retrospective case–control study, nor the funding or logistics to conduct a methodologically better prospective cohort study. If the recruitment of study participants to a cross‐sectional is principally amongst individuals of which some are assumed or known to have a particular risk or a protective factor, and the aim is to establish the prevalence of ill‐health amongst the study participants, the study is termed prospective cross‐sectional. An alternative is to recruit amongst individuals where some are more likely or known to have ill‐health (“cases”), with an objective to establish whether any adverse events or exposures have occurred recently or currently, and the study is described as retrospective cross‐sectional. If applied to cross‐sectional studies on peri‐implantitis in implant dentistry, the inference would be active recruitment of study participants with a history of periodontitis to estimate the prevalence of peri‐implantitis would become a “prospective cross‐sectional study”. The corollary is to recruit study participants with peri‐implantitis to establish if, for example, they smoke or have been smokers or have never smoked, and labelling such study as a “retrospective cross‐sectional study.” Some will state that these additional terms are redundant as long as the paper contains a proper description of the inclusion and exclusion criteria of the study participant relative to the pool of potential participants and with further details about the actual recruitment process and drop‐outs.

A substantial number of the 1,166 papers that adds the temporal element to “cross‐sectional” include the term incorrectly upon scrutinizing the description in the materials and methods section. Of the 32 papers in the dental journals, one paper described the changing prevalences of oral mucosal lesions amongst HIV/AIDS patients over time (Zakrzewska & Atkin, 2003). The majority of papers were cross‐sectional studies that alluded to the recruitment process, whereas others were case series or retrospective case–control studies where the authors had access to all previous patient data. About one fifth of the titles did not reflect the description of the study design described in the materials and methods section, and the reason for including the temporal element in the title remains obscure.

The terminology of clinical study designs has changed over time and is likely to continue to change. A contemporary title today that include “double‐blind randomized controlled trial” seems to offer more value for the potential reader compared with the less pretentious titles of early double‐blind RCTs, for example, “clinical trial” (Amberson, McMahon, & Pinner, 1931) and “controlled investigation” (U. K. Medical Research Council, 1948). A prudent approach for future authors is to adopt the newest terminology used in Medline for describing the study design and publication type to avoid confusing the readers. Moreover, pay attention to describing where and how the recruitment process for study participation was carried out and ensure that all inclusion and exclusion criteria are detailed. Finally, adhere to the many excellent recommendations for best reporting of health research detailed on the website of the equator network (www.equator‐network.com), of which some recommend the inclusion of the study design in the title of the paper. The gain will be avoiding several hours of languishing labor responding to referees that may ask uneasy questions about the accuracy of use of study design terminology.

## References

[cre2211-bib-0001] Altman, D. G. (1991). Practical statistics for medical research: Douglas G. Altman (p. 441). London: Chapman and Hall.

[cre2211-bib-0002] Amberson, J. B. , McMahon, B. T. , & Pinner, M. (1931). A clinical trial of sanocrysin in pulmonary tuberculosis. American Review of Tuberculosis, 24, 401–435.

[cre2211-bib-0003] Bacchieri, A. , & Della Cioppa, G. (2007). Fundamentals of clinical research: Bridging medicine, statistics and operations (p. 29). New York: Springer Science & Business Media.

[cre2211-bib-0004] Bradford Hill, A. (1971). Principles in medical statistics. Chapter XX. Clinical trials & Chapter XXI In Common fallacies and difficulties (9th ed.) (pp. 244–285). London, U.K.: The Lancet Ltd.

[cre2211-bib-0005] Brunette, D. (1996). Critical thinking In Understanding and evaluating dental research (p. 125). Chicago: Quintessence.

[cre2211-bib-0006] Celentano, D. D. , & Szklo, M. (2018). Gordis epidemiology (6th ed.) (p. 174). Philadelphia, P.A.: Elsevier Health Sciences.

[cre2211-bib-0007] Dekkers, O. M. , Egger, M. , Altman, D. G. , & Vandenbroucke, J. P. (2012). Distinguishing case series from cohort studies. Annals of Internal Medicine, 156(1 Pt 1), 37–40. 10.7326/0003-4819-156-1-201201030-00006 22213493

[cre2211-bib-0008] Esene, I. N. , Ngu, J. , El Zoghby, M. , Solaroglu, I. , Sikod, A. M. , Kotb, A. , … El Hussein, H. (2014). Case series and descriptive cohort studies in neurosurgery: The confusion and solution. Childs Nervous System, 30, 1321–1332. 10.1007/s00381-014-2460-1 24938735

[cre2211-bib-0009] Fletcher, R. H. , & Fletcher, S. W. (2013). Clinical epidemiology: The essentials (5th ed.). Baltimore: Lippincott Williams & Wilkins.

[cre2211-bib-0010] Gordis, L. (1996). Epidemiology (p. 139). Philadelphia: W.B. Saunders Comp.

[cre2211-bib-0011] Groten, M. , Janda, R. , & Latta, M. (2004). Clinical investigations of medical devices in dentistry: Specification, interpretation, and practical guidance (p. 78). Berlin, Germany: Quintessence Publishing Company.

[cre2211-bib-0012] Guyatt, G. , Sackett, D. , & Haynes, R. B. (2006). Evaluating diagnostic tests In Clinical epidemiology: how to do clinical practice research (p. 181). Baltimore: Lippincott Williams & Wilkins.

[cre2211-bib-0013] Hulley, S. B. , & Cummings, S. R. (1988). Designing clinical research (p. 64). Baltimore: William & Wilkins.

[cre2211-bib-0014] NCI – National Cancer institute . (2019). Dictionary of cancer terms. URL: https://www.cancer.gov/publications/dictionaries/cancer‐terms?expand=C

[cre2211-bib-0015] Pocock, S. J. (1983). Clinical trials: A practical approach (p. 51). Ames: John Wiley & Sons.

[cre2211-bib-0016] Sargeant, J. M. , O'Connor, A. M. , Cullen, J. N. , Makielski, K. M. , & Jones‐Bitton, A. (2017). What's in a name? The incorrect use of case series as a study design label in studies involving dogs and cats. Journal of Veterinary Internal Medicine, 31, 1035–1042. 10.1111/jvim.14741 28544149 PMC5508368

[cre2211-bib-0017] U.K. Medical Research Council (1948). Streptomycin treatment of pulmonary tuberculosis. British Medical Journal, 2(4582), 769–782. 18890300 PMC2091872

[cre2211-bib-0018] Zakrzewska, J. M. , & Atkin, P. A. (2003). Oral mucosal lesions in a UK HIV/AIDS oral medicine clinic. A nine year, cross‐sectional, prospective study. Oral Health and Preventive Dentistry, 1, 73–79. 10.3290/j.ohpd.a8223 15643751

